# Experimental demonstration of the novel “van-Hove integral method (vHI)” for measuring diffusive dynamics by elastic neutron scattering

**DOI:** 10.1038/s41598-021-93463-7

**Published:** 2021-07-08

**Authors:** Antonio Benedetto, Gordon J. Kearley

**Affiliations:** 1 School of Physics, University College Dublin, Dublin 4, Ireland; 2 Conway Institute, University College Dublin, Dublin 4, Ireland; 3 Department of Sciences, University of Roma Tre, Rome, Italy; 4 Laboratory for Neutron Scattering, Paul Scherrer Institute, Villigen, Switzerland; 5 School of Chemistry, University College Dublin, Dublin 4, Ireland

**Keywords:** Physics, Techniques and instrumentation

## Abstract

Quasi-elastic neutron scattering (QENS)—based on the seminal work of Nobel Laureate Brockhouse—has been one of the major methods for studying pico-second to nano-second diffusive dynamics over the past 70 years. This is regarded as an “inelastic” method for dynamics. In contrast, we recently proposed a new neutron-scattering method for dynamics, which uses the elastic line of the scattering to access system dynamics directly in the time domain (Benedetto and Kearley in Sci Rep 9:11284, 2019). This new method has been denoted “vHI” that stands for “van Hove Integral”. The reason is that, under certain conditions, the measured elastic intensity corresponds to the running-time integral of the intermediate scattering function, $I\left( {Q,t} \right)$, up to a time that is inversely proportional to the energy band-width incident on the sample. As a result, $I\left( {Q,t} \right)$ is accessed from the time derivative of the measured vHI profile. vHI has been supported by numerical and Monte-Carlo simulations, but has been difficult to validate experimentally due to the lack of a suitable instrument. Here we show that vHI works in practice, which we achieved by using a simple modification to the standard QENS backscattering spectrometer methodology. Basically, we varied the neutron-energy band-widths incident at the sample via a step-wise variation of the frequency of the monochromator Doppler-drive. This provides a measurement of the vHI profile at the detectors. The same instrument and sample were also used in standard QENS mode for comparison. The intermediate scattering functions, $I\left( {Q,t} \right)$, obtained by the two methods—vHI and QENS—are strikingly similar providing a direct experimental validation of the vHI method. Perhaps surprisingly, the counting statistics of the two methods are comparable even though the instrument used was expressly designed for QENS. This shows that the methodology modification adopted here can be used in practice to access vHI profiles at many of the backscattering spectrometers worldwide. We also show that partial integrations of the measured QENS spectrum cannot provide the vHI profile, which clarifies a common misconception. At the same time, we show a novel approach which does access $I\left( {Q,t} \right)$ from QENS spectra.

Pico-to-nanoseconds dynamical processes play a key role in nanotechnology and living systems. For example, in biophysics, the dynamics of enzymes and phospholipids play an important role in their biochemical function^1–4^. As a result, several methods have been developed and optimized over the last century to access these systems’ dynamics^5–10^. Among those, neutron-scattering approaches, in particular, have a few unique advantages^11–19^. These include (i) the ability of neutrons to interact directly with the nuclei of the atoms, which also facilitates comparison with molecular dynamics simulations; (ii) sensitivity to light atoms, particularly hydrogen; (iii) ability to distinguish between isotopes, and (iv) an innate molecular spatial resolution. Among neutron techniques, quasi-elastic neutron scattering (QENS) is the major approach for diffusive dynamics^20–24^. QENS instruments are found at every neutron scattering large scale facility^25–40^, serving a large and heterogeneous scientific community of users^41–60^.

At atomic and molecular level, the dynamical information can be mathematically represented by time-dependent probability-distribution functions^61^. The *self* (or autocorrelation) distribution function, $g\left( {r,t} \right)$, represents the probability that a particle composing the system—e.g. one of its atoms—moves by a distance *r* in a time *t*, and it is the one used to describe diffusion-type processes of interest here. Its spatial Fourier transform (FT) gives the *intermediate scattering function*, $I\left( {Q,t} \right)$, and its spatial-time-FT gives the *scattering function*, $S\left( {Q,E} \right)$, that is normally measured in inelastic and quasi-elastic scattering. In a scattering process, the $E$ value is the energy transfer, $E = \hbar \omega$, with $\omega$ being the Fourier inverse variable of *t*. The *Q* value, instead, is associated with the momentum transfer; it represents the Fourier inverse variable of *r* and is related to the scattering angle. $I\left( {Q,t} \right)$ and $S\left( {Q,E} \right)$ clearly contain the same system-dynamics information, and theoretically one can be obtained from the other by FT, as sketched in Fig. 1. However, with the finite limitations of practical measurements, the time and the energy (or frequency) points-of-views are not equivalent in experiments. This is because the FT operation needs to use all the *t*-points to get each individual $E$-point, and correspondingly, all the $E$-points are required to compute the $I\left( {Q,t} \right)$ at each single *t*-point. Crucially, the aim of many experiments for dynamics is access to access $I\left( {Q,t} \right)$, but only $S\left( {Q,E} \right)$ is actually measured.

**Figure 1:**
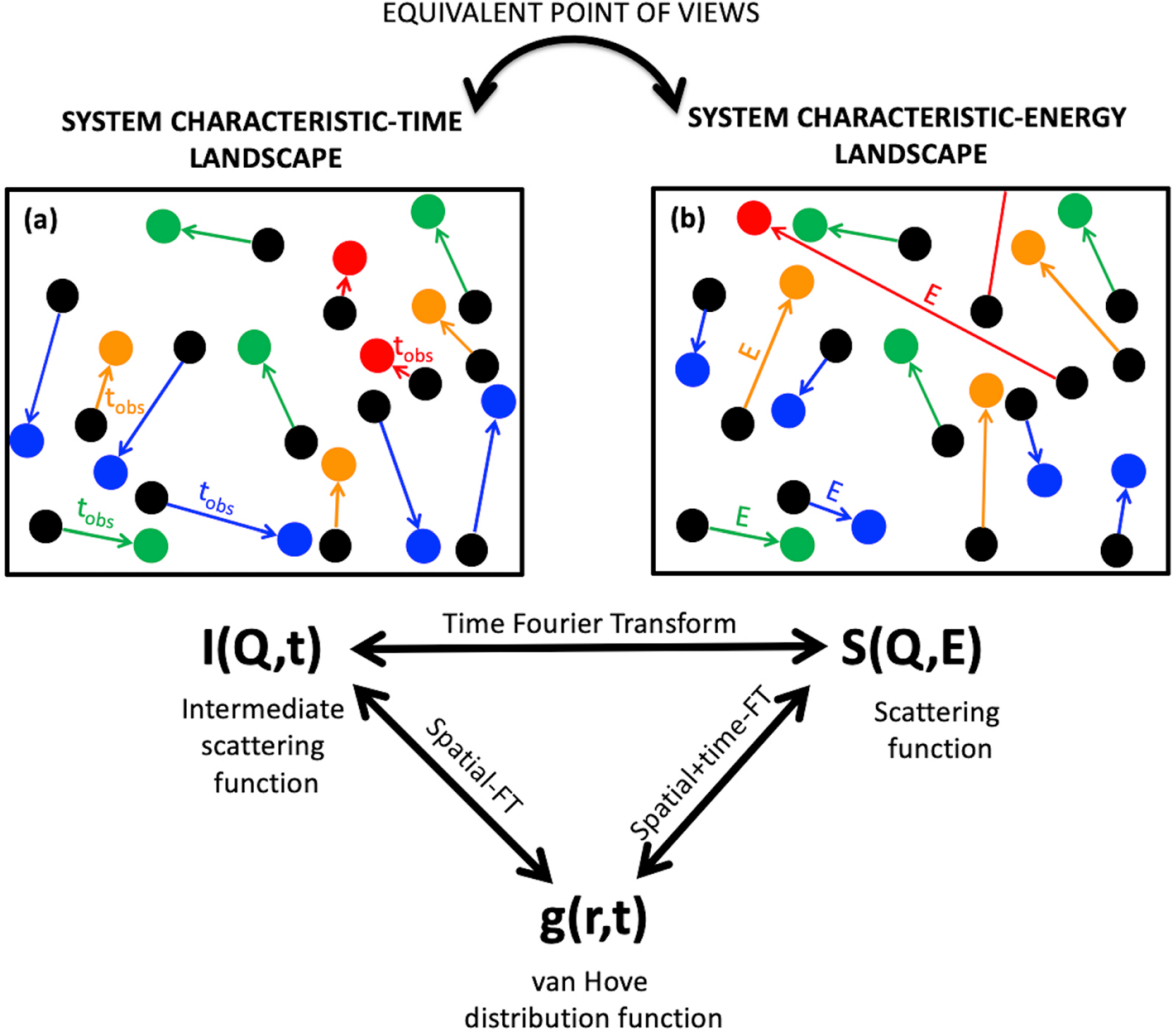
System (**a**) characteristic-time and (**b**) characteristic-energy landscapes for diffusive processes with the associated distribution functions linked together by FT operations. The characteristic time of a system relaxation process, $t_{{obs}}$, is inversely proportional to the characteristic energy, $E$.

QENS, based on the seminal work of Brockhouse^62^, measures the distribution of the energy-exchanges between the neutron and the sample over a range of different scattering angles. This distribution is known as “QENS spectrum” and usually is referred to as the “measured scattering function, $S_{R} \left( {Q,E} \right)$”. The accessible energy-exchange, ranging from tens of micro-eV to hundreds of milli-eV, depends on several instrumental features. The information on the diffusive dynamics is contained in the spectral profile around the zero-energy exchange value—known as the *elastic line*—rather than in inelastic features. $S_{R} \left( {Q,E} \right)$ is the convolution of the system scattering function, $S\left( {Q,E} \right)$, with the instrumental energy resolution function, $R\left( {Q,E} \right)$. $R\left( {Q,E} \right)$ contains the instrumental distortions and limits the observation of slower dynamical processes. It is usually measured either by using a “pure incoherent elastic scatterer”, such as vanadium, or by measuring the sample at a temperature low enough to eliminate dynamics at the time-scale accessible by the instrument. Often, $R\left( {Q,E} \right)$ is approximately Gaussian in shape and its width is known as *instrumental energy resolution*, $dE_{{Res}}$. The measured spectrum $S_{R} \left( {Q,E} \right)$ needs to be corrected to take proper account of these instrumental features. This can be achieved via the numerical time-FT of $S_{R} \left( {Q,E} \right)$ divided by the numerical time-FT of $R\left( {Q,E} \right)$, which gives the system $I\left( {Q,t} \right)$ and it is reported in Eq. (1). In summary, QENS is a method that accesses system dynamics by measuring energy exchanges, and so can be described as an “inelastic method for dynamics”.

1 $$S_{R} \left( {Q,E} \right) = S\left( {Q,E} \right) \otimes R\left( {Q,E} \right) \to I\left( {Q,t} \right) = \frac{{time - FT\left\{ {S_{R} \left( {Q,E} \right)} \right\}}}{{time - FT\left\{ {R\left( {Q,E} \right)} \right\}}}.$$

Recently, prompted by a few previous attempts^63–79^, we have proposed a new neutron scattering method for diffusive dynamics, which has been denoted “vHI” that stands for “van Hove Integral”^80^. Our vHI method measures the neutrons scattered into the elastic line as a function of the observation time, $t_{{obs}}$, which is inversely proportional to the neutron-energy band-width incident at the sample. The basis of our method is that, under certain conditions, the vHI profile is the running-time integral of the system $I\left( {Q,t} \right)$. As a result, $I\left( {Q,t} \right)$ is accessed from the time derivative of the measured vHI profile, as summarized in Eq. (2). With adequate counting statistics this is a simple numerical differentiation but this is achieved, in practice, with the polynomial derivative method^80^. In summary, vHI is a method that accesses system dynamics by measuring the solely elastic line component of the scattering, and so it can be described as an “elastic method for dynamics”. This is the major conceptual difference in respect to the QENS approach, providing a more straightforward way to the investigation of dynamical diffusive processes.

2 $$vHI\left( {Q,t_{{obs}} } \right) = \mathop \smallint \limits_{0}^{{t_{{obs}} }} I\left( {Q,t} \right)dt \to I\left( {Q,t} \right) = \frac{d}{{dt_{{obs}} }}vHI\left( {Q,t_{{obs}} } \right).$$

vHI has been formulated theoretically and proven by numerical and Monte-Carlo simulations^80,81^; it is now accepted as valid. Here we present the first experimental proof of the method’s validity. Since there are no spectrometers designed for vHI, this experimental test has been performed by modifying the procedure of a standard QENS high-resolution backscattering spectrometer to work in vHI mode. We recently used a similar procedure in a Monte-Carlo simulation of a neutron backscattering instrument that was equally capable of operating in either vHI or QENS mode^81^. Here we compare the $I\left( {Q,t} \right)$-function obtained in vHI mode, $I_{{vHI}} \left( {Q,t} \right)$, with that from the QENS mode, $I_{{QENS}} \left( {Q,t} \right)$. The details on how a standard QENS spectrometer has been used in vHI mode are presented in the “methodology” section at the bottom of this letter. The protocol on how the $I\left( {Q,t} \right)$ has been extracted from the measured vHI profile is also presented in the “methodology” section along with the more standard procedure to extract it from standard QENS. In the “methodology” section we also show that the vHI profile cannot be computed from the QENS spectrum, clarifying a common misunderstanding.

The experiment was carried out on the IN16B high-resolution QENS backscattering spectrometer^25^ at the Institute Laue-Langevin (ILL), Grenoble, France, using Ferrocene^82–84^ as a sample. The experiment was run at 130 K where the quasi-elastic component is known to be resolved by IN16B. To take account of instrumental features, both vHI profile and QENS spectrum were also collected at 2 K where no measurable dynamical processes are occurring in the system^85^.

To judge the accuracy of vHI for $I\left( {Q,t} \right)$, we have compared the $I_{{vHI}} \left( {Q,t} \right)$ with the $I_{{QENS}} \left( {Q,t} \right)$. Figure 2 shows the $I_{{vHI}} \left( {Q,t} \right)$ and the $I_{{QENS}} \left( {Q,t} \right)$ summed at several scattering angles. Even casual inspection confirms that the functions are very similar. The smoothness of the two functions does not reflect the statistical-scatter of the experimental data, but this happens for different reasons. For QENS, the FT operation effectively filters frequencies and mixes *x* and *y* errors, resulting in a strong smoothing. Although for vHI the derivative can be obtained numerically (no smoothing), it is more convenient to use the polynomial derivative protocol^80^, which then gives a smooth $I\left( {Q,t} \right)$, analogous to that obtained from the FT of QENS. A single exponential function plus a flat background is able to fit the $I\left( {Q,t} \right)$-functions adequately. The relaxation time obtained by the vHI method results 5% shorter than the relaxation times obtained with QENS.

**Figure 2:**
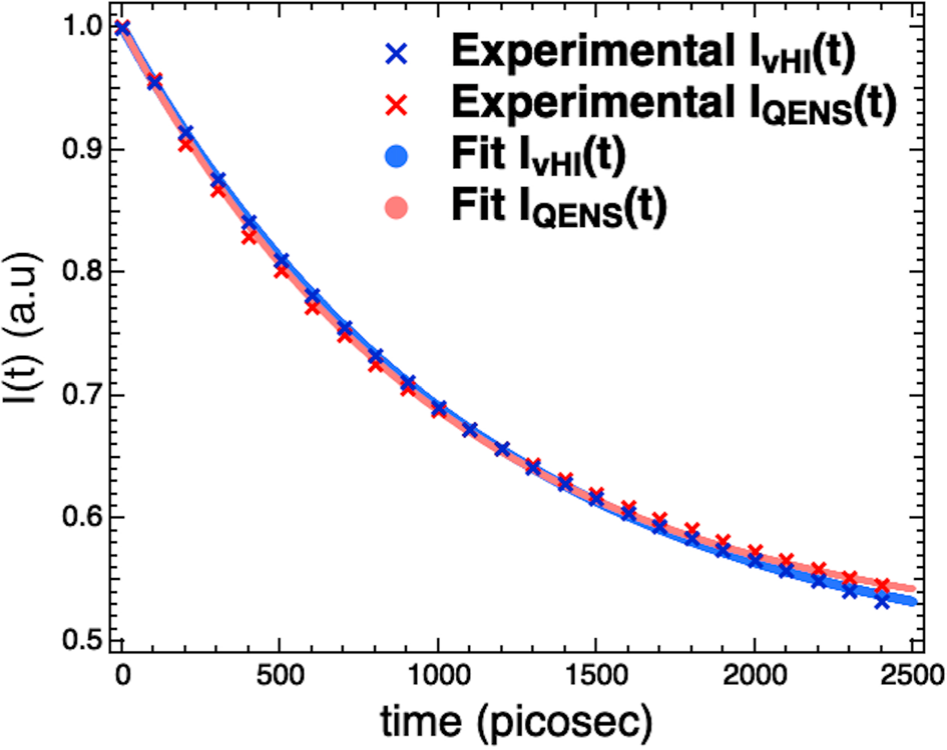
Comparison between $I_{{vHI}} \left( {Q,t} \right)$ (blue) and $I_{{QENS}} \left( {Q,t} \right)$ (red) summed at several scattering angles.

The results in Fig. 2 are the sum at several scattering angles and allow us to compare vHI and QENS with the best counting statistics from the experimental data. At reduced statistical significance, we can go further and make comparisons at each scattering angle. For this, each $I\left( {Q,t} \right)$ has been fitted with an exponential function plus a flat background, and the resulting relaxation times have been plotted in Fig. 3 as function of *Q*. Because the scattered neutron energy-range is small compared with the incident energy, there is effectively no difference between QENS and vHI for the *Q*-dependence. Effectively, these twelve Q-values can be considered as simply twelve independent experiments, each confirming that vHI reproduces the QENS result. The relaxation times obtained by vHI are in line with QENS confirming, in turn, that the vHI method works in practice.

**Figure 3:**
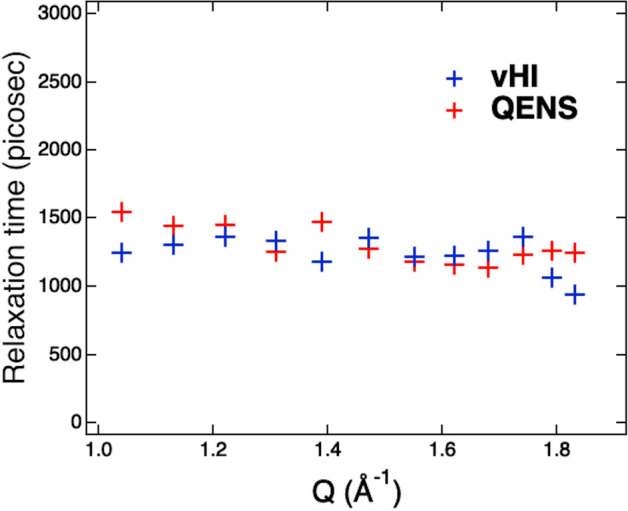
Relaxation times obtained by fitting $I_{{vHI}} \left( {Q,t} \right)$ and $I_{{QENS}} \left( {Q,t} \right)$ with an exponential function plus a constant background for several Q values.

In conclusion, we have shown here that in a real experiment vHI provides the same information as QENS, notwithstanding that the instrument was designed for QENS. Use of different Doppler-drive frequencies is an effective means of mimicking different energy-bandwidths, suggesting that it can be used in practice to access vHI profiles at a number of backscattering spectrometers worldwide. Obviously, a purpose-built instrument would provide vastly better counting statistics. Surprisingly, however, the counting statistics of the two methods are comparable even though the instrument used was expressly designed for QENS. This is in line with our previous studies showing that vHI can achieve tenfold better statistics at $I\left( {Q,t} \right)$-level than QENS^81^, making the use in vHI-mode of the existing QENS backscattering spectrometers a feasible option for any interested users.

The results presented here complete the validation study of the vHI method—it is now tested and validated via theory, simulation and experiment. The stage is now set for instrumental layouts optimized for vHI. Advantages of vHI over QENS that can be explored in the design of a dedicated spectrometer include:

- Ideally, the improved counting statistics of vHI could be traded to access longer times, by using tighter monochromation methods that would otherwise be too expensive on count-rates^86^.
- vHI needs to measure the solely elastic line intensity, whereas QENS needs to compute each energy transfer. This should provide a much easier design for a dedicated vHI instrument.
- Further, since the elastic line intensity is a few orders of magnitude higher than the inelastic one, vHI seems to be much more suitable for low power and/or compact sources.
- Each time-point is measured independently in vHI. In QENS, instead, all the measured energy-points contribute to each time point as required by the FT operation. As a result, vHI (i) can have a different counting time at each time point (as actually done in this study), and (ii) it is not affected by either the truncation errors present in QENS due to the finite energy range of the experiment nor by the need to choose model fitting functions.

## Methodology

### QENS backscattering spectrometers in their “standard” QENS mode

**Figure 4:**
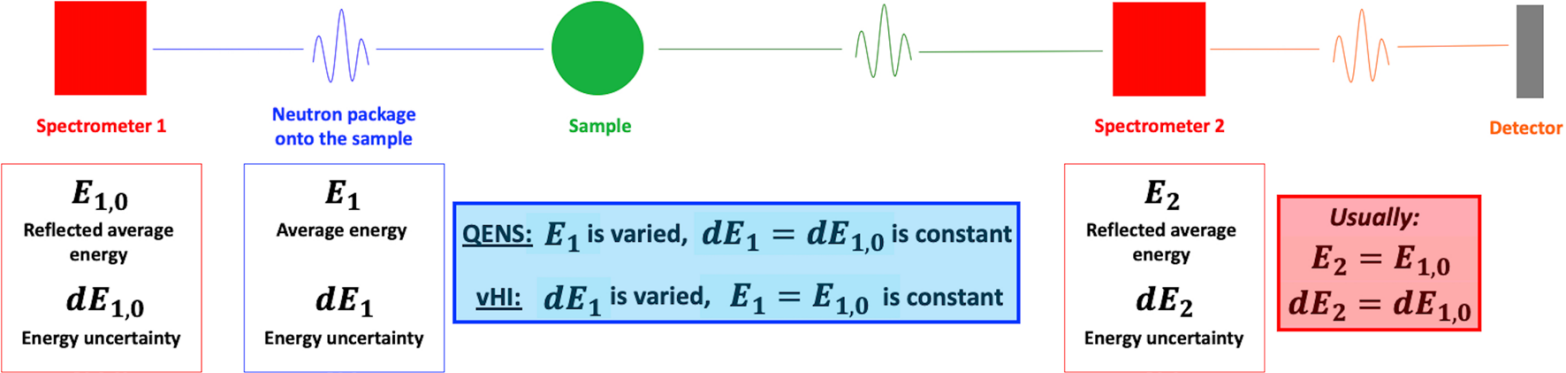
The most general layout and *modus operandi* of a neutron backscattering spectrometer in which the interplay between the primary and the secondary spectrometers enables the measurement of either the QENS spectrum or the vHI profile. QENS needs to compute the energy exchange $E$ due to the scattering process and thus operates in the “inelastic” regime with fixed energy resolution $dE_{{Res}} \approx \sqrt {dE_{{1,0}}^{2} + dE_{2}^{2} }$. vHI is fundamentally different, it operates in the purely “elastic” regime. For both QENS and vHI there are three constant quantitates and one variable. Here, for QENS, dE_1_, dE_2_ and E_2_ are constant and E_1_ is varied (with, usually, dE_1_ = dE_2_ resulting in $dE_{{Res}} \approx 1.4 \cdot dE_{1}$). For vHI, instead, E_1_, E_2_ and dE_2_ are constant and dE_1_ is varied, (with E_1_ = E_2_ and $dE_{2} \le dE_{1}$).

In standard QENS mode the spectrometer works as follows (Fig. 4). The neutron beam from the neutron guide impinges on a crystal monochromator mounted on a Doppler drive in an almost backscattering geometry. This portion of the instrument is usually referred to as “primary spectrometer”. Only the neutrons with energies matching the energy-band of the crystal shifted by the Doppler-drive velocity are reflected onto the sample. At any instant, this package of reflected neutrons has energies distributed following a Gaussian-like distribution, with a constant energy band-width, $dE_{1}$, equal to the energy uncertainty of the crystal monochromator, $dE_{{1,0}}$. The average energy of the reflected neutron, instead, is shifted due to the Doppler effect from the average energy reflected by the crystal monochromator, $E_{{1,0}}$, to $E_{1} \left( {v_{{Doppler}} } \right)$, function of the speed of the Doppler-drive. These neutrons are scattered by the sample where they can exchange both energy and momentum. Scattered neutrons are analysed by a set of crystal analyzers that are usually the same as that on the Doppler, i.e. $E_{2} = E_{{1,0}}$ and $dE_{2} = dE_{{1,0}}$. In this way only the scattered neutrons with energies $E_{{1,0}} \pm dE_{{1,0}}$ are reflected to the detectors, providing an overall energy uncertainty of $dE_{{Res}} \approx \sqrt {dE_{{1,0}}^{2} + dE_{2}^{2} } = 1.4 \cdot dE_{{1,0}}$, known as *instrumental energy resolution*. If the Doppler is at rest, only elastically scattered neutrons can arrive at the detectors. On the contrary, for the Doppler-drive moving at speed $v_{{Doppler}}$, only the neutrons that exchange an energy $E$ such as $E = E_{1} \left( {v_{{Doppler}} } \right) - E_{{1,0}}$ can be detected. By correlating the Doppler velocity with the arrival time of neutrons at the detector, the energy exchange can be determined. Analyzers at different scattering angles provide spectra at different *Q*-values. As a result, the $S_{R} \left( {Q,E} \right)$ is measured.

### vHI mode for QENS backscattering spectrometers

To work in vHI mode, we adapted the operation of a standard QENS backscattering spectrometer as follows (Fig. 4). With the Doppler-drive at rest the energy and associated band-width of the neutrons from the first spectrometer is $E_{{1,0}} \pm dE_{{1,0}}$. The analysers reflect exactly the same energies (being $E_{2} = E_{{1,0}}$ and $dE_{2} = dE_{{1,0}}$), giving a total count at the detectors, $N_{{detectors}} \left( {Q,dE_{{1,0}} } \right)$, for solely elastic intensity. This is what vHI is supposed to measure, which corresponds to the running-time integral of $I\left( {Q,t} \right)$ computed up to $t_{{obs}} = 1.37/dE_{{1,0}}$ (corrected for Gaussian resolution functions) times the number of incoming neutrons from the first spectrometer, $N_{{in}} \left( {dE_{{1,0}} } \right)$, times the associated energy band-width $dE_{{1,0}}$^80^. With the Doppler-drive oscillating at an angular frequency $\omega _{{Doppler}}$, within a complete oscillation cycle, a distribution of neutron energies centred around $E_{{1,0}}$ is generated due to the Doppler effect. The energy band-width depends on $\omega _{{Doppler}}$, i.e. $dE_{1} \left( {\omega _{{Doppler}} } \right)$. It collapses to $dE_{{1,0}}$ for $\omega _{{Doppler}} = 0$ and it increases by increasing $\omega _{{Doppler}}$. As a result, the distribution of neutron energies at the sample is always centred at $E_{{1,0}}$ and its band-width can be controlled by changing the frequency of the Doppler-drive, with $dE_{{1,0}}$ being its lowest value achievable with the Doppler-drive at rest. This energy band-width-controlled neutron package is then scattered by the sample to the crystal analysers, which only reflect neutrons with energies of $E_{{1,0}} \pm dE_{{1,0}}$ (being $E_{2} = E_{{1,0}}$ and $dE_{2} = dE_{{1,0}}$). As a result, the total count at the detectors, $N_{{detectors}} \left( {Q,dE_{1} \left( {\omega _{{Doppler}} } \right)} \right)$, corresponds to the running-time integral of $I\left( {Q,t} \right)$ computed up to $t_{{obs}} = 1.37/dE_{1} \left( {\omega _{{Doppler}} } \right)$ times the number of incoming neutrons from the first spectrometer,${\text{~}}N_{{in}} \left( {dE_{1} \left( {\omega _{{Doppler}} } \right)} \right)$, times the associated energy band-width, $dE_{1} \left( {\omega _{{Doppler}} } \right)$, as reported below in Eq. (3):^80^

3 $$N_{{detectors}} \left( {Q,dE_{1} \left( {\omega _{{Doppler}} } \right)} \right) = N_{{in}} \left( {dE_{1} \left( {\omega _{{Doppler}} } \right)} \right) \cdot dE_{1} \left( {\omega _{{Doppler}} } \right) \cdot \mathop \smallint \limits_{0}^{{t_{{obs}} }} I\left( {Q,t} \right)dt$$

in which the running time integral of $I\left( {Q,t} \right)$ is the $vHI\left( {Q,t_{{obs}} } \right)$ profile itself. As a result, to compute $vHI(t_{{obs}} )$, the spectrum at the detector has been (i) divided by the corresponding monitor spectrum to both normalize for $N_{{in}}$ and correct, at the same time, for the U-shaped energy distribution generated by the Doppler-drive, (ii) divided by $dE_{1} \left( {\omega _{{Doppler}} } \right)$, and (iii) integrated in energy-transfer. By using the backscattering spectrometer in this mode, the vHI profile can be measured by varying the $\omega _{{Doppler}}$, with each $\omega _{{Doppler}}$ being associated to a different value of $t_{{obs}}$. Note that Eq. (3) has been rigorously obtained by us in the *Supplementary Information* of Ref.^80^. Briefly, this has been done by assuming that the integral of the energy distribution of the neutron package generated by the first spectrometer corresponds to the number of incoming neutrons into the sample, which has to be kept the same for each acquisition at the different $\omega _{{Doppler}}$; sample and secondary spectrometer, instead, have been described by probability distribution functions normalized to the unity. In summary, a standard backscattering spectrometer can be used in vHI mode by simply operating the primary spectrometer in a different way (Fig. 4). This modification to the standard QENS methodology can be used in practice, as we did here, to access vHI profiles at a number of backscattering spectrometers worldwide representing, *the facto*, the first operative VHI spectrometers.

### The vHI experiment

**Figure 5:**
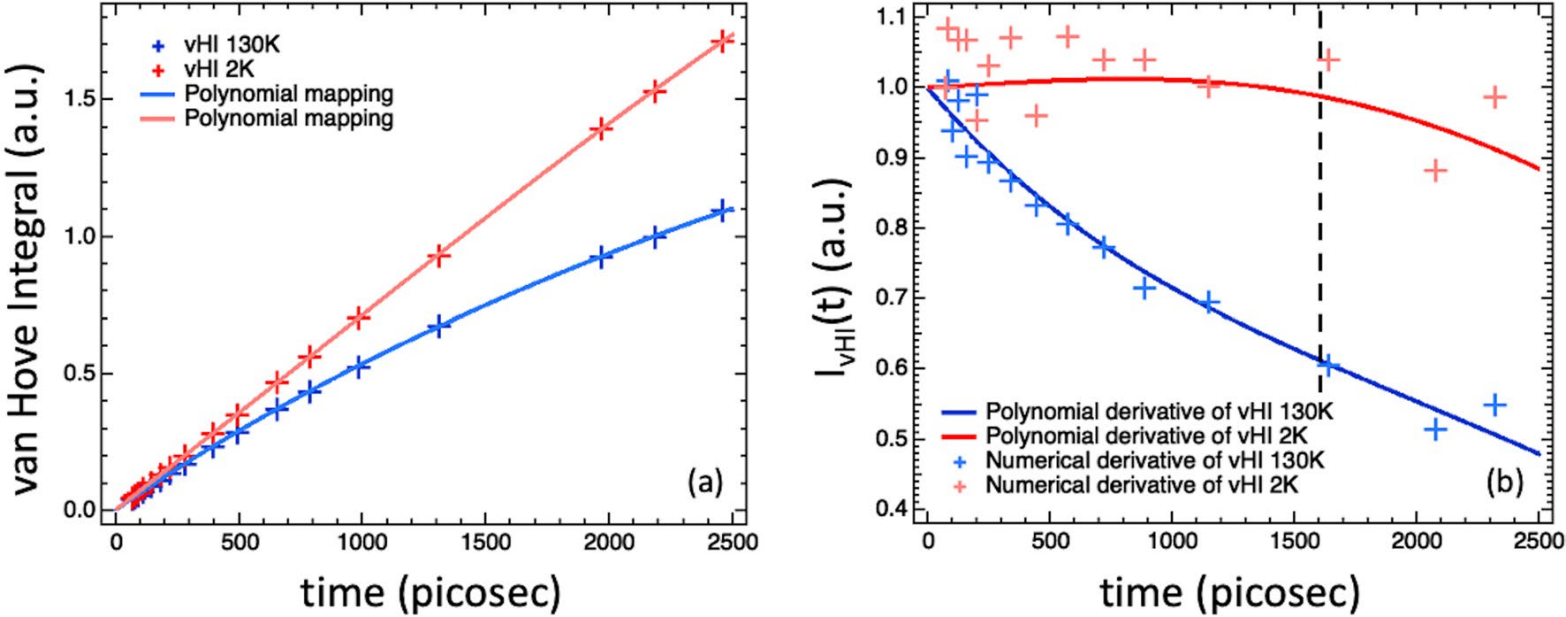
(**a**) vHI profiles at 130 K and 2 K summed over several *Q*-values with the associated polynomial mapping, and (**b**) their time derivatives, obtained both numerical and by the polynomial derivative method. The dashed line in (**b**) indicates the longest $t_{{obs}}$ value accessible as imposed by the instrumental features of the used spectrometers which, however, can be extended to 2500 ps by “correcting” for the vHI 2 K run as presented at the bottom of the methodology section.

In vHI-mode, twenty independent acquisitions at twenty different Doppler-frequencies were used to measure the vHI profile. They covered an incoming neutron energy-width range from approximately $0.3$ µeV (Doppler at rest) to $30$ µeV (highest frequency), which corresponds to $t_{{obs}}$ spanning two orders of magnitude from about 30 to 3000 ps. The longest accessible time resulted, however, limited to 1600 ps by the by instrumental features, including the energy-band width of the second spectrometer (0.5 µeV). As for QENS, however, also for vHI the 2 K run can be used to extend the accessible time widow to 2500 ps, as presented at the bottom of this methodology section. The acquisition times were two minutes for the first ten points (lower $\omega _{{Doppler}}$) and then increased to have approximately the same number of total counts per run (up to twelve minutes for the highest $\omega _{{Doppler}}$ corresponding to $dE_{1} = 30$ µeV). In total, collecting the vHI profile took two and a half hours. The sample temperature was 130 K, with an analogous measurement at 2 K being made to take account of instrumental features. The results are shown in Fig. 5a, where the unequal spacing of the points in $t_{{obs}}$ arises from measuring at incremental energy-spacing, rather than incremental $t_{{obs}}$ that would be used in future experiments. To access the system $I\left( {Q,t} \right)$ the time derivative of the 130 K vHI profile has been computed. The time derivative of the 2 K vHI profile, on the other hand, was used to compute the longest $t_{{obs}}$ accessible by the instrument as the $t_{{obs}}$ from which it diverges from unity. The results are shown in Fig. 5b. It is encouraging that the numerical derivative of the two vHI profiles is more tractable than expected, and that their fits (not shown) work quite well. The more stable polynomial derivative method, selected as the standard protocol, results in curves that resemble the best fits of the numerical derivatives (which they are not). We suggest that in a dedicated vHI spectrometer, numerical derivatives would be available, but in general these would not offer any advantage over the more efficient polynomial derivation method.

### The QENS experiment

**Figure 6:**
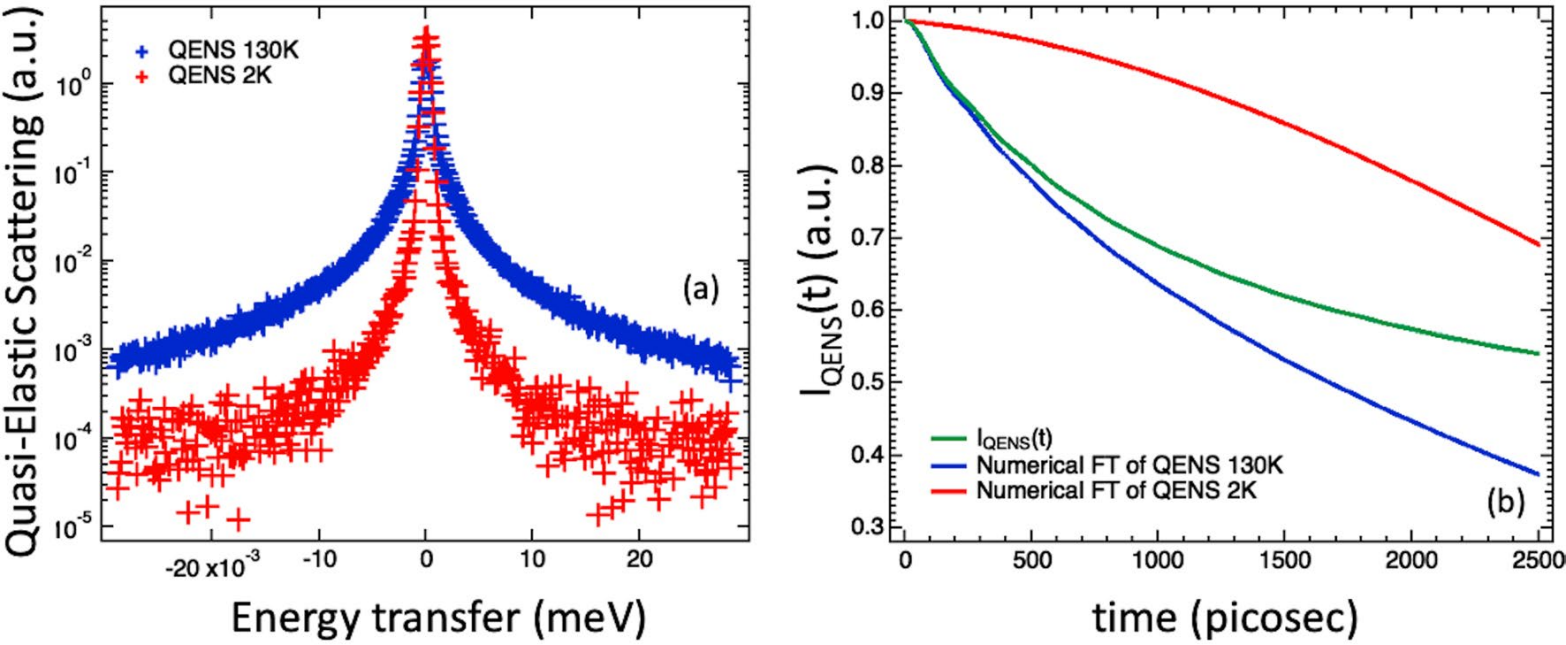
(**a**) QENS spectra at 130 K and 2 K, and (**b**) the associated numerical time-FT along with their ratio to compute the $I_{{QENS}} \left( {Q,t} \right)$, summed over several *Q*-values.

Whereas in our previous computational works^80,81^ we were able to compare the $I_{{vHI}} \left( {Q,t} \right)$ with the input-$I\left( {Q,t} \right)$, this is obviously not possible here. To judge the accuracy of the vHI method in accessing $I\left( {Q,t} \right)$, we compared it with the $I_{{QENS}} \left( {Q,t} \right)$ obtained by carrying out a standard QENS experiment on the same instrument. IN16B was used in its standard mode $\left( {E_{{max}} = 30\,\upmu {\text{eV}},dE_{{Res}} = 0.5\,\upmu {\text{eV}}} \right)$. The counting time was three hours that is comparable with the two and a half hours used for the vHI profile. The QENS instrument has a clear counting-advantage in QENS mode, but nevertheless, when using comparable acquisition times, the counting statistics of the two techniques are actually comparable. The measurement was made at 130 K, with an analogous measurement at 2 K to characterize the instrumental resolution. The results are shown in Fig. 6a. To access the system $I\left( {Q,t} \right)$, the measured QENS needs to be “corrected” by the instrumental resolution function. As outlined in the introduction, the $I_{{QENS}} \left( {Q,t} \right)$ was obtained by dividing the numerical time-FT of the 130 K-QENS spectrum by the numerical time-FT of the 2 K-QENS spectrum. The results are shown in Fig. 6b.

### Three methodological remarks

**Figure 7:**
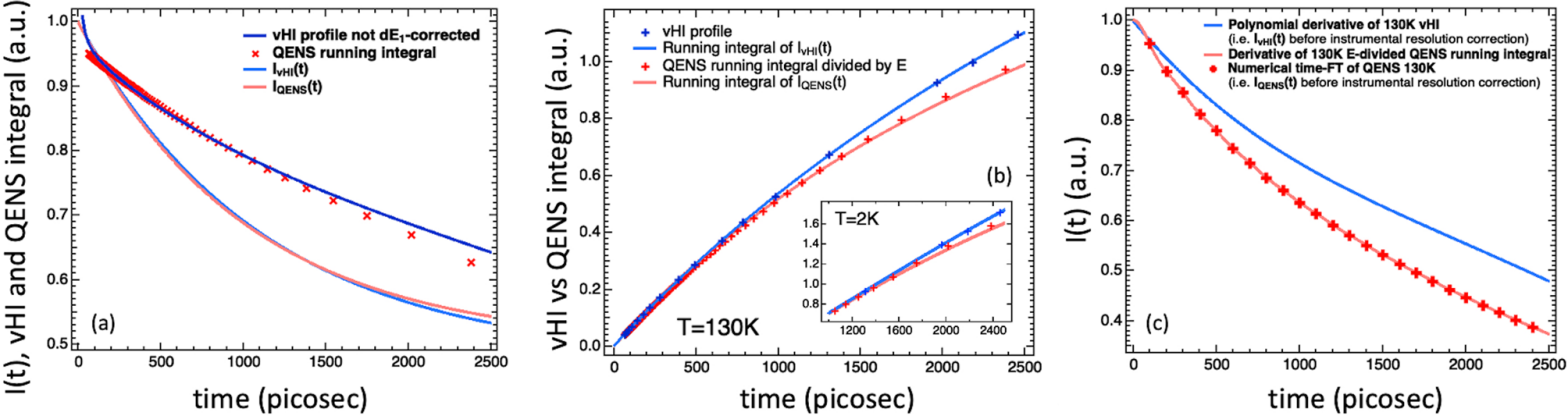
(**a**) Comparison between the $E$-running integral of the measured QENS spectrum, the measured vHI profile not dE_1_-corrected, and the system intermediate scattering function at 130 K. (**b**) Comparison between the $E$-running integral of the measured QENS spectrum divided by $E$ and the measured vHI profile at 130 K. The time-running integrals of I_vHI_(t) and I_QENS_(t), before the instrumental resolution correction, are also shown. The inset reports the case for 2 K. (**c**) Comparison between the derivatives of the integrals in (**b**) clearly showing that the two approaches give different outcomes, so they are not equivalent. The time-FT of the 130 K QENS spectrum is also shown.

The first remark is about the misconception that the measured elastic intensity corresponds directly to the intermediate scattering function evaluated at $t_{{obs}}$^63^. By comparing the intermediate scattering function and the vHI profile (not normalized by $dE_{1}$), Fig. 7a clearly shows that, even though trends of these two functions are similar, this earlier assumption is wrong, as already anticipated by our vHI theory^80^.

**Figure 8:**
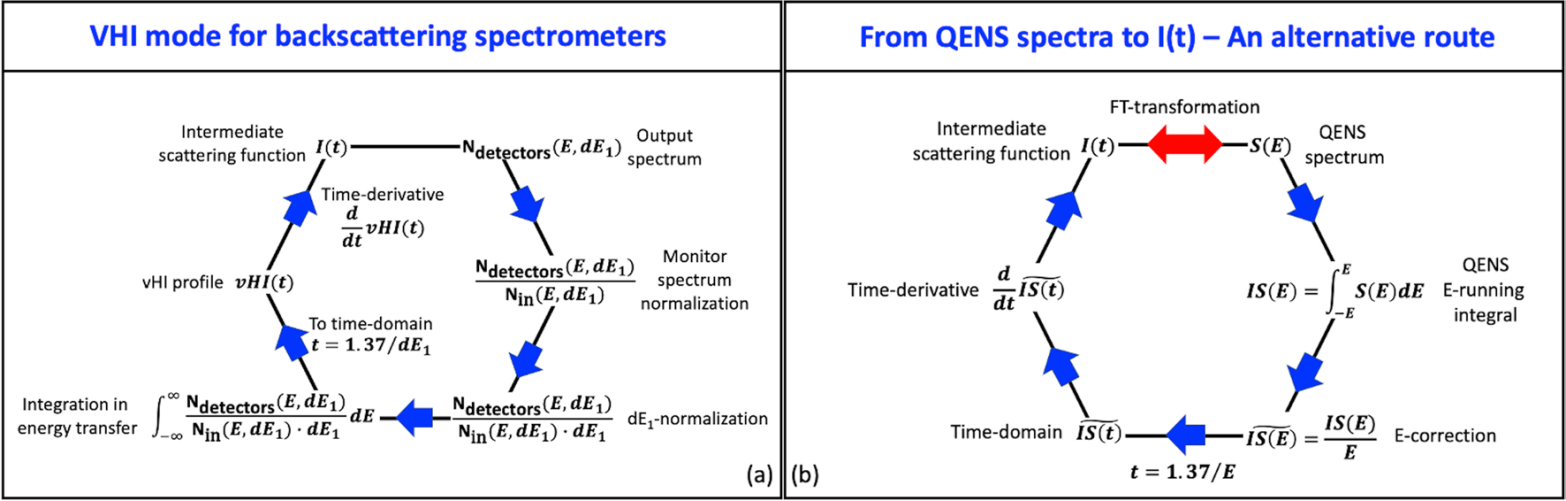
(**a**) Scheme showing the different steps for the vHI mode of a standard QENS backscattering spectrometer to access the vHI profile from the measured output and then compute I(t). (**b**) Scheme showing the different steps of an alternative method to access I(t) from the measured QENS spectrum. The well-known FT-transformation is also reported. The factor in the observation time-energy transfer relationship, depends on the shape of the neutron package energy distribution from the first spectrometer; the value of 1.37 is valid for backscattering spectrometers employing a Doppler-drive. In practice, it can be computed theoretically or determined from a calibrated standard sample.

The second remark is about the general misunderstanding that the vHI profile could be obtained by partial integrations of the QENS spectrum. Figure 7a shows the numerical energy transfer-running integral of the measured QENS spectrum along with the vHI profile not normalized by $dE_{1}$ (see Eq. 3). The QENS $E$-running integral has been plotted in the time-domain by using the $t_{{obs}} = 1.37/E$ vHI-relationship for the observation time, with the difference that now the role of the incoming energy band-width is played by the running value of the QENS energy transfer, $E$, (i.e. the limit of the running QENS integral) rather than by $dE_{1}$ as in the vHI case. Figure 7b reports the measured vHI profile (normalized by $dE_{1}$) along with the numerical $E$-running integral of the measured QENS spectrum divided by the associated $E$ value (i.e. equivalent to multiplying by $t_{{obs}}$ in the time domain). Even though the trend is similar, it can be seen, by looking at Fig. 7a,b, that these two profiles do not overlap in either case. Figure 7c shows their time derivatives resulting in two different $I\left( {Q,t} \right)$ functions, clearly showing that vHI cannot be obtained by integrating QENS. Intuitively, the rationale behind this is as follows. In a QENS backscattering experiment the distribution of neutron energies fired into the sample accounts for all the measurable energies (i.e. the energy window). This is not the case for vHI, where this distribution of energies is changing during the acquisition. As a result, QENS and vHI are two different experiments. It can be shown that they overlap in the ideal case of delta instrumental energy resolution functions. Notably, the time derivative of the $E$-divided QENS partial numerical $E$-integrations, gives $I_{{QENS}} \left( {Q,t} \right)$ before the resolution correction, i.e. the time-FT of the measured QENS spectrum (Fig. 7c). On the other hand, (i) the running integral of the $I_{{QENS}} \left( {Q,t} \right)$ gives the $E$-divided QENS partial numerical $E$-running integral, and (ii) the running integral of the $I_{{vHI}} \left( {Q,t} \right)$ gives the vHI profile, i.e. the neutron counts at the detectors, in vHI-mode, normalized by the number of incoming neutrons from the first spectrometer and divided by dE_1_ (Fig. 7b). This correspondence shows the importance/meaning of the dE1-correction of Eq. (3) and opens the way to a novel data analysis route to get $I\left( {Q,t} \right)$ from QENS spectra (of diffusive motions), which has the advantages of being (i) model free and (ii) unaffected of truncation errors, occurring in standard QENS approaches to $I\left( {Q,t} \right)$ routinely used. Figure 8 reports a schematic summary of both vHI and QENS methodologies for I(t).

**Figure 9:**
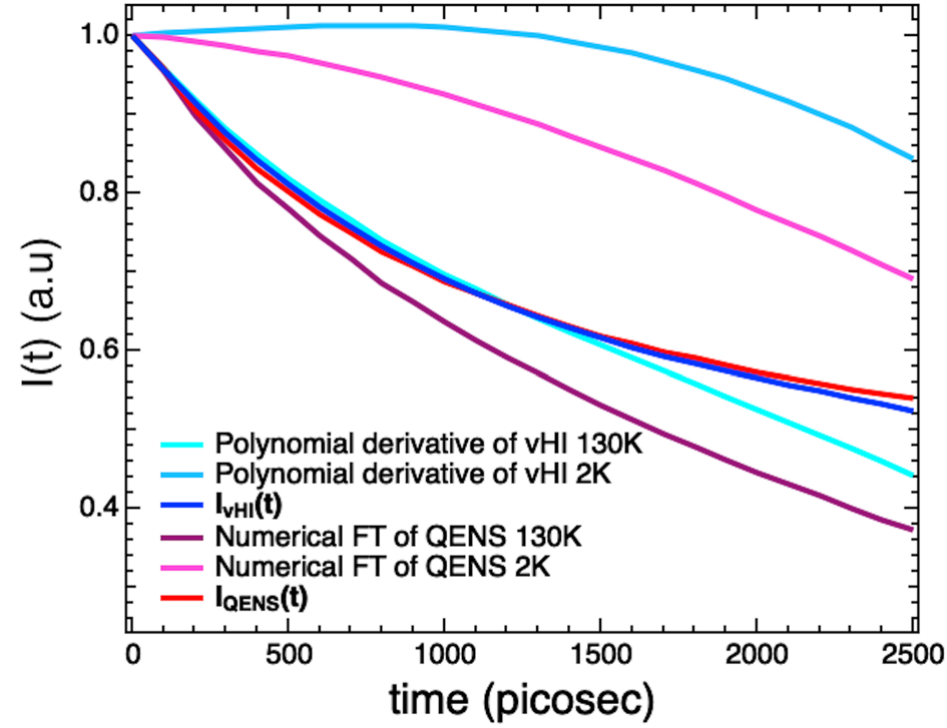
(**a**) Comparison between the “corrected” $I_{{vHI}} \left( {Q,t} \right)$ (blue) and $I_{{QENS}} \left( {Q,t} \right)$ (red) together with all the functions involved in vHI and QENS corrections, summed at several scattering angles.

The final remark is about pushing the boundary of vHI towards longer times. To access the full experimental time range, similar considerations and corrections to those for QENS can be applied. In this case, the vHI profile of either a perfect elastic scatterer or the sample at a very low temperature can be collected and then used to correct the measured $I\left( {Q,t} \right)$. The correction consists in dividing the time derivative of the measured vHI profile with the time derivative of this elastic-scatterer vHI profile to get the “corrected” $I_{{vHI}} \left( {Q,t} \right)$ of the system. As a result, to access the “corrected” $I_{{vHI}} \left( {Q,t} \right)$ the time derivative of the 130 K vHI profile has been divided by the time derivative of the 2 K vHI profile. The result is shown in Fig. 9. Notably, the 130 K and “corrected” $I_{{vHI}} \left( {Q,t} \right)$-functions perfectly overlap up to 1600 ps, which corresponds to the longest-time limit imposed by the instrumental features; this is not the case for the 130 K and “corrected” $I_{{QENS}} \left( {Q,t} \right)$-functions, which overlap for few tens of picoseconds only. This “corrected” $I_{{vHI}} \left( {Q,t} \right)$ has been used in the main text.
